# In Vitro Bioactivity of Leaf Extract Fractions and Quercetin-3-*O*-Rhamnoside from *Combretum elaeagnoides* Against *Staphylococcus* Species Implicated in Causing Bovine Mastitis

**DOI:** 10.3390/ijms27031579

**Published:** 2026-02-05

**Authors:** Rosemary Chinelo Erhabor, Jean Paul Dzoyem, Inge-Marie Petzer, Muna A. Abdalla, Lyndy Joy McGaw

**Affiliations:** 1 Phytomedicine Programme, Department of Paraclinical Sciences, Faculty of Veterinary Science, University of Pretoria, Private Bag X04, Onderstepoort, Pretoria 0110, South Africa; 2 Department of Biochemistry, Faculty of Science, University of Dschang, Dschang P.O. Box 96, Cameroon; 3 Department of Production Animal Studies, Faculty of Veterinary Sciences, University of Pretoria, Private Bag X04, Onderstepoort, Pretoria 0110, South Africa; 4 Institute of Plant Nutrition and Soil Science, University of Kiel, Hermann-Rodewald−Str. 2, 24118 Kiel, Germany

**Keywords:** bovine mastitis, *Combretum elaeagnoides*, biofilm, quorum sensing, *Staphylococcus aureus*, anti-inflammatory, antibiotic resistance, quercetin-3-*O*-rhamnoside

## Abstract

Globally, antibiotic resistance is a growing concern, motivating the search for alternatives. Bovine mastitis is an inflammatory disease of the udder caused by various microorganisms, many of which are resistant to various antibiotics, impacting the quality of dairy products and farmer income. In this study, the in vitro bioactivity of the methanol leaf extract, fractions (ethyl acetate (CeEtOAc), butanol (CeBuOH), hexane (CeHx), dichloromethane CeDCM), and water (CeAq), and a purified compound, quercetin-3-*O*-rhamnoside isolated from the CeEtOAc fraction of *Combretum elaeagnoides* Klotzsch, were investigated against six *Staphylococcus aureus* (*S. aureus*) strains isolated from clinical cases of bovine mastitis and two reference ATCC strains (*S. aureus* ATCC 29213 and *S. epidermidis* ATCC 35984). Methods used for assessing bioactivity included serial microdilution for antibacterial efficacy, crystal violet staining and *p*-iodonitrotetrazolium (INT) metabolic assays for anti-biofilm activity, and a microdilution assay for anti-quorum-sensing potential. The anti-inflammatory assays included 15-lipoxygenase enzyme inhibition and nitric oxide assays. Cytotoxicity screening was conducted using a tetrazolium-based colorimetric assay against bovine dermis cells. The extracts and fractions exhibited moderate to good antibacterial activity with minimum inhibitory concentration (MIC) values ranging from 0.07 to 1.04 mg/mL, with the ethyl acetate fraction being the most effective. The anti-biofilm activity of the extract, fractions, and isolated compound (quercetin-3-*O*-rhamnoside) varied at time zero (T0), with inhibition ranging from 3% to 100%. The CeDCM and CeEtOAc fractions exhibited the most potent anti-biofilm effects after 24 h, with inhibition ranging from 24% to 91%. The extracts and fractions exhibited significant inhibition (>50%) of biofilm within the incubation times (T0–T48), and quercetin-3-*O*-rhamnoside alone had >60% inhibition at 48 h. The CeEtOAc fraction had the most significant anti-quorum-sensing activity (IC_50_ < 0.08 mg/mL). The methanol extract and fractions exhibited significant anti-inflammatory activity, inhibiting nitric oxide production (IC_50_: 7–26 µg/mL). In contrast, the CeAq, CeHx, and CeDCM fractions showed the best inhibitory activity against the 15-lipoxygenase enzyme (IC_50_ = 3–4 µg/mL). The extracts and fractions were non-cytotoxic to bovine dermis cells (LC_50_ = 0.88–1 mg/mL). *Combretum elaeagnoides* extract and its fractions are recommended for further investigation as potential herbal treatments for the management of mastitis and its symptoms.

## 1. Introduction

Bovine mastitis is the most important dairy disease and accounts for the highest antibiotic use in dairy farming [1]. Bovine mastitis is a recurring and communicable infection that could have a direct or indirect impact on the dairy cow and its products, resulting in economic losses [2,3]. The direct impact of bovine mastitis on the chemical and physical condition of milk results in milk wastage and increased treatment costs. The indirect impact of bovine mastitis can reduce milk quantity and quality and may require culling [2]. *Staphylococcus aureus* (*S. aureus*) is a contagious pathogen that can be found living in the udder and on the teat skin of cows, with the ability to colonise the teat canal, resulting in a mammary parenchyma infection [4]. Although *S. aureus* can be considered a low-priority problem in the dairy industry in some countries, it remains a major challenge in South African dairy herds, as resistance to antibiotics is increasing [3,5].

Inflammation can be classified into two types: acute and chronic inflammation. Acute inflammation is the body’s first mechanism for responding to injury or infection, characterised by redness, swelling, warmth, and pain around the affected area. This helps to accelerate the healing process [6]. According to Harvard Health (2020), chronic inflammation has been attributed to life-threatening diseases, e.g., cardiovascular disease, cancer, type 2 diabetes, and other conditions in humans [6]. Chronic inflammation is a prolonged inflammation with a tendency to recur and has been associated with bovine mastitis cases, especially in clinical cases of bovine mastitis [4], especially when *S. aureus* is involved. Inflammation of the udder tissue (bovine mastitis) in cows can lead to embryo loss and a decreased pregnancy rate [7].

Bovine mastitis (BM) is classified into two different forms: The first is the clinical cases, which are associated with swelling, pain, watery milk, dehydration, loss of appetite, and fever. Cows with clinical cases of BM usually have an increased somatic cell count greater than 200,000 cells/mL [8]. In contrast, subclinical cases of BM have no visible signs of infection but result in reduced milk production and quality [8].

Dairy farms may be a source of antimicrobial-resistant bacteria that are pathogenic to humans, posing a significant health hazard. Antibiotic residues in milk can disrupt consumers’ intestinal flora, potentially leading to allergic reactions and contributing to antimicrobial resistance [3,5,9]. The rise in microbial resistance to antibiotics and multi-drug resistance (MDR) has increased mortality rates and the cost of medical treatment for infectious diseases [10,11]. Antibiotic resistance can occur through microbial biofilm formation, which can cause modifications to the host cell, allowing the organisms to survive as sessile populations within an enclosed exopolysaccharide, additionally, limiting antibiotic penetration and promotes a metabolically inactive bacterial state less susceptible to antimicrobial action [12,13]. Biofilm formation promotes microbial survival and increases disease virulence, usually initiated by microbial quorum sensing. Continuous genetic variation and spontaneous mutations in microbial populations have significantly contributed to the rapid emergence and spread of antimicrobial resistance (AMR) [14,15]. Notable examples include methicillin-resistant *Staphylococcus aureus* (MRSA) and vancomycin-resistant enterococci (VRE). This results from the acquisition of the *mecA* or *mecC* gene, which encodes an alternative penicillin-binding protein (PBP2a) exhibiting low affinity for β-lactam antibiotics, enabling cell wall synthesis to proceed despite antibiotic exposure. The production of *β*-lactamase enzymes by *S. aureus* hydrolyses the β-lactam ring of penicillin antibiotics, which can lead to the inactivation of the drug [15]. Efflux pumps such as NorA can also lead to antimicrobial resistance actively expelling antibiotics such as fluoroquinolones from the bacterial cell, reducing intracellular drug concentrations [16]. These resistance traits are frequently acquired and disseminated through horizontal gene transfer mediated by plasmids, transposons, and staphylococcal cassette chromosome mec (SCCmec) elements [10,17,18].

Medicinal plants have demonstrated significant pharmacological activities against a wide range of infections and pathogenic strains over the years. They have proven to possess a wide range of secondary bioactive metabolites effective against microbial infections [19,20,21,22,23,24,25]. Plant species belonging to the Combretaceae family are among the most widely used medicinal plants in African traditional medicine [26,27]. In southern Africa, there are over 300 species of *Combretum*. The family Combretaceae is native to Africa, tropical Asia, America, and Madagascar [28]. The family has been reported to have a wide range of pharmacological bioactive constituents with antibiofilm, quorum quenching, anti-inflammation, antifungal, antiprotozoal, antitumor, antiviral, and antibacterial potential, as well as wound healing properties [26,27,29,30]. *Combretum elaeagnoides* Klotzsch is one of the understudied species of the Combretaceae family and is native to Botswana, Zimbabwe, the Caprivi Strip, Zambia, and Mozambique [22,31]. Leaves of this deciduous tree are used in traditional medicine in Zambia to treat diarrhoea, tuberculosis, skin infections, malaria and sexually transmitted infections (STIs) [32,33]. This species had noteworthy activity against some food-borne pathogens [22] and was therefore selected for this study.

## 2. Results and Discussion

### 2.1. Minimum Inhibitory Concentration (MIC)

**Figure 1:**
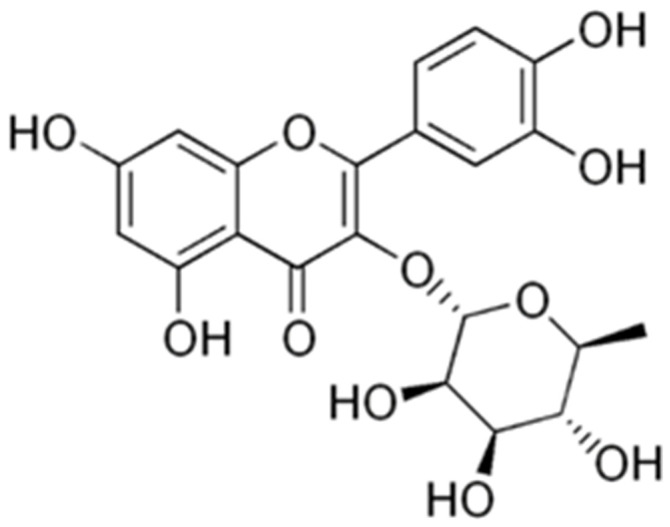
The structure of quercetin-3-*O*-rhamnoside (quercitrin).

The in vitro susceptibility activities of each fraction and the methanol extract tested are shown in Table 1. Erhabor et al. (2021) reported on the antibacterial activity of the methanol extract and different solvent fractions as well as the isolated compound (quercetin-3-*O*-rhamnoside, Figure 1) from *Combretum elaeagnoides* leaves against various strains of foodborne pathogens [22]. The CeEtOAc fraction had a lower MIC against various bacteria implicated in causing food-borne diseases compared to the other samples. Corroborating these results, the CeEtOAc fraction had the overall best broad-spectrum activity against all *S. aureus* strains (MIC 0.07–0.23 mg/mL) in the present study. The compound quercetin-3-*O*-rhamnoside also exhibited good MIC values (0.31–1.25 mg/mL) against *S. aureus*, comparable to its activity against the food-borne pathogens (MIC = 1.25–2.5 mg/mL) previously reported [22]. Although most of the samples exhibited significant antibacterial activity against the pathogens, the positive control (gentamicin) had a more potent activity when compared with the samples. The abundant presence of quercetin-3-*O*-rhamnoside in the CeEtOAc fraction may have contributed to or enhanced the activity of the CeEtOAc [22]. Further research is needed to quantify the amount of the compound in the extract, and also to isolate further potentially active compounds. The fractions and isolated compound quercetin-3-*O*-rhamnoside had better inhibition against the test pathogens than the crude methanol extract. The effects of the different fractions, methanol extract, and quercetin-3-*O*-rhamnoside against *S. aureus* showed significant activity compared with their activity against food-borne pathogens and may be considered for further screening to implement their use in managing bovine mastitis cases. Several reports have been published on the effect of *Combretum* species against bovine mastitis, with a particular focus on *Combretum molle*. Although *C. elaeagnoides* is investigated for the first time against bovine mastitis, it exhibited potent antibacterial activity, which correlates with the antibacterial effect of *Combretum* spp. [34,35].

### 2.2. Anti-Biofilm Activity

#### 2.2.1. Inhibition of Cell Attachment and Biofilm Biomass Formation

The effect of methanol extracts, fractions, and a purified compound on *S. aureus* showed varying levels of inhibition of cell attachment (anti-adhesion T0), biofilm formation (T24) and the disruption of mature biofilm (T48) (Table 2 and Table 3). The antibiofilm activity of the leaf methanol extracts, different fractions and the compound against some foodborne pathogens (*Salmonella enterica* subsp. *enterica* serotype Typhimurium (*Salmonella* Typhimurium) ATCC 39183 [38], *Salmonella* Enteritidis ATCC 13076 [39], *Escherichia coli* 1 ATCC 25922 [40], *Escherichia coli* 2 (clinical isolate), *Staphylococcus aureus* ATCC 29213, *Campylobacter jejuni* ATCC 33560 [41], *Stenotrophomonas maltophilia* (clinical isolate), *Klebsiella pneumoniae* (clinical isolate) and *Enterobacter cloacae* (clinical isolate)) tested at time zero (T0) was previously reported [22]. In that report, the different fractions of *C. elaeagnoides* and compound (quercetin-3-*O*-rhamnoside) showed selective antibiofilm activity against the different food-borne pathogens. The results of this study against mastitis-causing organisms are reported for the first time and correlate with the findings of the samples against food-borne pathogens. Although *C. molle* has been reported to inhibit biofilm formation against bovine mastitis pathogens [30], the results obtained in the study showed that different extracts of *C. molle* had good antibiofilm activity against SA1 and SA3 when compared with the current study. The isolated compound (1 mg/mL) was tested only after 48 h of incubation, due to the limited amount available. Three of the fractions (CeDCM, CeEtOAc, and CeHx) showed the best antiadhesion activity against all test pathogens (34–100% inhibition) at T0, with CeHx having >50% inhibition against all pathogens. The strain SA4 was completely susceptible to the CeHx fraction at all periods of incubation. The samples showed selective inhibition against preformed biofilm biomass at T24 and matured biofilm at T48; most samples showed reduced or no inhibition against the pathogens at T48, and the increased bacterial aggregation might have reduced the effect of the samples, which limits adequate penetration of the samples. The CeDCM and CeBuOH fractions exhibited antibiofilm activity against four of the test pathogens; however, the CeEtOAc fractions showed the greatest activity (>50%) at T24 against five of the pathogens. After 48 h of biofilm formation, the CeHx fraction alone reduced and inhibited preformed biofilms on three pathogens, with inhibition > 50%.

Interestingly, the DCM fraction against SA3 exhibited good antibiofilm activity when compared to the positive controls. Likewise, a similar result was obtained for the hexane fraction against SA4, and most of the sample against SEA. At T48, quercetin-3-*O*-rhamnoside alone showed good antibiofilm activity (˃50%) in five test strains (SA1, SA4, SA5, SEA, and SAA). It also had good activity against five of the pathogens compared to the positive control (gentamicin). *Combretum* species are common medicinal plants with a wide range of bioactive compounds [19,20]. For instance, a recent study found that the extract of *Combretum micranthum* G. Don inhibited the biofilm formation of *Pseudomonas aeruginosa* and its isogenic biofilm hyperproducer derivative B13 [42]. Consequently, this confirms the potential of *Combretum* species to counter mechanisms that enhance bacterial survival and resistance to antimicrobial agents, thereby enabling the efficient activity of microbial agents by targeting pathogens’ defence mechanisms, thereby reducing their ability to multiply. For further progress, the fractions in this study could be investigated to isolate more specific bioactive compounds, especially from the methanol extract, which could also be responsible for the activity.

**Figure 2:**
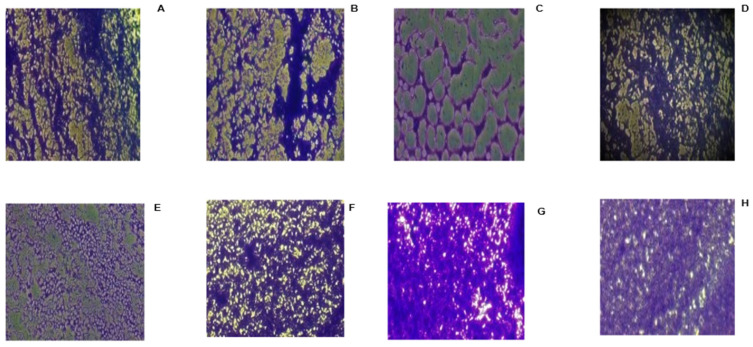
The microscopic image of the biofilm-forming level/ability of the different pathogens after 24 h of incubation and staining with crystal violet. The purple spots indicate the formation of biofilm or cell attachment on the 96-well plates. ((**A**) = *S. aureus* clinical isolate 1; (**B**) = *S. aureus* clinical isolate 2; (**C**) = *S. aureus* clinical isolate 3; (**D**) = *S. aureus* clinical isolate 4; (**E**) = *S. aureus* clinical isolate 5; (**F**) = *S. aureus* clinical isolate 6; (**G**) = *S. aureus* ATCC 29213; (**H**) = *S. epidermis* ATCC 35984).

The current study examined whether the studied bacteria are biofilm formers. The organisms were cultured for 24 h, following several steps as described in Section 3.4.1. After staining with crystal violet, the plates were examined under a microscope to assess their biofilm structure (Figure 2). Several imaging techniques have been used in previous biofilm studies, which increased the in-depth understanding of their structure [43]. The different pathogens showed varying levels of biofilm formation under the microscope. The aggregation of pathogens on the 96-well microplate, retaining the crystal violet stain, demonstrated that the pathogens could form a good biofilm. Additionally, after quantifying the biofilm by resolubilising the plate with 98% ethanol, the biofilm OD measured with the microplate reader ranged from 2.165 to 3.715 nm, which is more than 10 times that of the standardised bacterial culture. According to Adeyemo et al. (2022) [44], bacteria are considered good biofilm formers if the mean OD of the test pathogen is 4 times greater than the OD of the culture control. All pathogens could attach and form biofilms on the plate (Figure 2), which were viewed using the trinocular Nikon Eclipse TS100 optical microscope. However, *S. aureus* ATCC 29213 exhibited the highest biofilm-forming ability. The biofilms were evenly distributed at the walls and bottom of the plate, with the highest confluency, followed by *S. epidermidis* ATCC 35984 and the *S. aureus* clinical isolate. This was used to determine the antibiofilm activity of the samples.

#### 2.2.2. Evaluation of the Biofilm Metabolic Activity of the Different Fractions and Extracts of *C. elaeagnoides*

Biofilm metabolic inhibitory activity of *C. elaeagnoides* is reported for the first time. Biofilms are known to occur in a different form in the presence of nutrients that promote bacterial growth and help them adjust to changes. The nutrients involved in biofilm formation enhance the metabolic activity of the organisms [45,46]. The methanol crude extract, different fractions, and compounds were tested against the metabolic/respiratory activity of the different pathogens in their biofilm form. The isolated compound was tested only at time T48 owing to insufficient quantities isolated, and showed good inhibition against the pathogens’ metabolic activity, with inhibition > 50% against all pathogens. The compound exhibited significant inhibitory activity against all pathogens (except SA1 and SA5) compared with the positive control, gentamicin, and against all pathogens (except SA3) when compared with ciprofloxacin.

Interestingly, all samples showed strong inhibition of the pathogens’ metabolic activity, especially the compound, which demonstrated good activity (bactericidal effect) against all pathogens when compared to the positive controls at T48. This means that the samples not only inhibited biofilm formation but also exerted a bactericidal effect on the pathogens in their biofilm form. The percentage of inhibition of the samples against the metabolic activity of the pathogens in their planktonic stage (T0) ranged from 31% to 100% (Table 4). The metabolic activity of the samples against the pathogens in their biofilm form (T24 and T48) exhibited varying levels of inhibition (3% to 100%). Although all samples showed good inhibition at T24 against most pathogens, the Me0H extract, CeEtOAc, CeH, and CeDCM fractions had the highest inhibition (>50%) against all pathogens. There was a reduction in the effect of the samples on the metabolic activity of the mature biofilm formed by the pathogens after 48 h of incubation; nevertheless, CeEtOAc showed excellent inhibition (>50%) against seven of the pathogens. The methanol extract and CeDCM exhibited excellent (>50%) inhibition against six pathogens, while CeBuOH and CeAq showed inhibition against five pathogens.

The ability of the samples to inhibit the metabolic/respiratory activity in the pathogens explains their ability to reduce biofilm formation. This result also suggests that the samples are effective antimicrobial agents that could aid in reducing and managing bovine mastitis when compared with the positive controls.

### 2.3. Anti-Quorum-Sensing (QS) Activity of Combretum elaeagnoides Against Chromobacterium violaceum (% Inhibition in mg/mL)

The anti-quorum-sensing potential of *C. elaeagnoides* is reported for the first time, using the model organism *Chomobacterium violaceum*, which produces violacein pigment, a visible and quantifiable marker for cell-to-cell communication. The quercetin-3-*O*-rhamnoside was not tested due to the insufficient amount obtained. The mechanism of quorum sensing involves regulating virulence factor production, modulating gene expression, and modifying the organism’s metabolism, leading to biofilm formation [47]. Various reports have described the inhibition of quorum-sensing signalling molecules, which are crucial for QS gene regulation in *C. violaceum* [30,44,48,49]. In this study, the methanol extract and the different fractions of *C. elaeagnoides* showed concentration-dependent inhibition of violacein production, with the percentage of inhibition ranging from 28–96.32% (Table 5). At the highest concentration, all samples showed a similar trend of violacein inhibition to that of the positive controls. At the concentration of 0.31 to 2.5 mg/mL, all samples exhibited ≥50% inhibition of violacein production. However, the CeEtOAc fraction alone inhibited ≥50% violacein production at all concentrations (50.83% to 96.21%), with a MIC of 0.63 mg/mL and a MQSIC of 0.08 mg/mL. Although they showed good violacein inhibition, not all samples were able to inhibit the growth of *C. violaceum*. The CeBuOH and CeDCM fractions also had above 50% violacein inhibition at 0.16 mg/mL. Although all samples showed good violacein inhibition, only CeH and CeDCM inhibited the growth of *C. violaceum*. This finding indicates that all fractions and the MeOH extracts inhibited violacein production in *C. violaceum* and have the efficacy to control or eliminate antimicrobial resistance facilitated by microbial quorum sensing. This suggests that the sample may inhibit or suppress *C. violaceum*’s pigmentation and reduce its virulence, thereby enhancing the efficacy of treatment against resistant pathogens.

### 2.4. Anti-Inflammatory Activity of the Methanol Extract and Fractions of Combretum elaeagnoides Against 15-Lipoxygenase, and Nitric Oxide Inhibition

Elevated 15-lipoxygenase (15-LOX) activity has been linked to chronic inflammatory and oxidative responses and is also implicated in numerous diseases [50,51]. The production of nitric oxide (NO) regulates a wide range of physiological and pathological processes associated with cardiovascular and inflammatory diseases, resulting in tissue damage and other diseases [52]. The ability of the MeOH extract and fractions was evaluated against the 15-LOX enzyme and NO production in LPS-induced RAW 264.7 macrophages. The methanol extract and fractions (Table 6) exhibited good to excellent inhibition against the 15-LOX enzyme, with IC_50_ values ranging from 3 to 47 µg/mL. The CeHx, CeAq and CeDCM fractions had the best inhibition (3.40, 4.49 and 3.59 µg/mL, respectively) against 15-LOX when compared to the positive control (quercetin IC_50_ = 9.70 µg/mL). The NO inhibition assay using the macrophages showed that the methanol extract and fractions (Table 6) were non-toxic to the cell lines, even at the highest concentration (100 µg/mL), and showed good nitric oxide inhibition (7–26 µg/mL). Again, four of the samples had good anti-inflammatory activity when compared with the positive control. The samples were less toxic to the cells than the reference drug (quercetin), which was toxic at the highest concentration tested (100 µg/mL). It would be interesting to evaluate the activity of the plant samples against somatic cells found in the milk of cows affected with bovine mastitis. *Combretum* species are used in traditional medicine to treat inflammation-related diseases, and various reports have documented their anti-inflammatory activity [53,54].

Therefore, the activity of the methanol extract and fractions of *C. elaeagnoides* supports the claim of its use in ethnopharmacology. The anti-inflammatory activity may be related to the presence of bioactive secondary metabolites and is recommended for novel therapeutic formulations to manage inflammation in bovine mastitis. Moreover, this activity also supports the ethnopharmacological relevance of this species, as also indicated by [55,56].

### 2.5. Cytotoxicity of the Methanol Extract and Fractions of Combretum elaeagnoides

The methanol extract and fractions were non-toxic to bovine dermis cells in this study, with LC_50_ values greater than 0.08 mg/mL (Table 7). According to Zirihi et al. (2005), an extract is non-toxic if the LC_50_ is ≥0.02 mg/mL [57]. The cytotoxicity of the methanol extract and fractions against Vero African green monkey kidney cells was reported in our previous study [22]. The samples were relatively non-toxic to the cells, with IC_50_ values ranging from 0.022 to 0.05 mg/mL. All fractions were less cytotoxic to bovine dermis cells compared to Vero cells. The selectivity index (SI) was also used as a parameter to determine the safety of the extract and fractions, measured as the ratio of cytotoxicity (LC_50_) to antibacterial activity (LC_50_/MIC) [58]. An SI value greater than 1 indicates that a compound is less toxic to cells than to bacteria [22,59]. All samples had SI values greater than 1; however, the CeEtOAc and CeDCM fractions had the best broad-spectrum SI and are considered to be safer to the cells, with potent antibacterial activity. The methanol extract and its fractions may offer a promising pharmacological approach in the treatment of mastitis, following further in vivo trials.

## 3. Materials and Methods

All measurements, including the different concentrations of the samples and positive controls, are standard concentrations established in our laboratory for the different assays.

### 3.1. Plant Material

The preparation of the samples and isolation of the purified compound were reported in our previous study [22].

### 3.2. Preparation of Bacterial Culture

Six of the *S. aureus* strains were clinical isolates from bovine mastitis cases collected from different farms in South Africa (ethical permission number V121-16, University of Pretoria), and two *Staphylococcus* ATCC strains (*S. aureus* ATCC 29213 and *S. epidermidis* ATCC 35984) were obtained from the Milk Laboratory, Department of Production Animal Studies, Faculty of Veterinary Sciences, University of Pretoria. The method used to identify the *S. aureus cultures* is reported by [60]. Several media were used for the different assays, including Mueller-Hinton broth (MHB), Mueller-Hinton agar (MHA), Tryptic Soy broth (TSB), Tryptic Soy agar (TSA), and Luria–Bertani (LB) broth, for assessing minimum inhibitory concentration, antibiofilm, and anti-quorum activity, respectively. The bacteria, after being revived in MHA (Fluka, Seelze, Germany), were kept at 4 °C. Each bacterial culture was cultured in the appropriate media for 24 h at 37 °C in an incubator prior to each experiment.

### 3.3. Bioassay for Antibacterial Activity

#### 3.3.1. Susceptibility Testing (Minimum Inhibitory Concentration)

The MIC is the lowest concentration of the extracts at which the microorganism does not show visible growth or reveal inhibition of growth following incubation with a test substance. This was determined following the method of Eloff (1998) [61]. The extracts were tested at a starting concentration of 2.5 mg/mL (from a stock concentration of 10 mg/mL) in a 96-well microtitre plate and serially diluted two-fold with sterile water to obtain a final concentration of 0.02 mg/mL. Following this, 100 μL of the overnight bacterial culture (standardised by measuring the absorbance of the diluted culture at 560 nm relative to a McFarland No. 1 standard (3 × 10^8^ CFU/mL) was added to each well. The positive control, gentamicin, with a serially diluted concentration range of 0.5 and 0.004 mg/mL, was used as the reference drug for the assay. The microtiter plates were covered with lids, sealed with parafilm, and incubated overnight at 37 °C for at least 18 h. As an indicator of bacterial growth, 40 μL *p*-iodonitrotetrazolium chloride (INT) (Sigma, St. Louis, MO, USA, 0.2 mg/mL) dissolved in sterile distilled water was added to the wells and incubated at 37 °C for 1 h. The MIC values were recorded as the lowest extract concentration that inhibited bacterial growth, as indicated by a marked reduction in colour formation. The INT turns red-pink to formazan, indicating that bacterial growth is not inhibited.

#### 3.3.2. Anti-Biofilm Screening

##### Inhibition of Bacterial Cell Attachment and the Formation of Biofilm via the Crystal Violet Assay

The inhibition of biofilm biomass formation was assessed using the protocol reported by Sandasi et al. (2010) [62] with slight modification [22]. The experiment was performed at three different times: time zero (0), 24 h (T24), and 48 h (T48). Briefly, biofilm was allowed to form for 24 and 48 h to allow irreversible attachment for the T24 and T48 experiments, respectively. Biofilm production was achieved by aliquoting 100 μL of the respective culture (OD_590_ = 0.02, equivalent to 1.0 × 10^6^ CFU/mL) into wells of a sterile flat-bottomed 96-well microtitre plate and sealing with sealing tape. The plates were incubated for 24 and 48 h, respectively. Following incubation, 100 μL of the extracts (at a final concentration of 1 mg/mL from a stock of 2 mg/mL) and the respective controls were transferred to their designated wells and incubated for an additional 24 h at 37 °C without shaking. Appropriate control wells were included in the plate, including negative control (culture + media (TSB)), positive control (culture + TSB + antibiotic), sample control (sample + TSB), antibiotic control (antibiotic + TSB), and media control (TSB) for each test batch. After incubation, the crystal violet staining (CVS) assay was performed to assess biofilm biomass, or the biomass of adhered cells. For the CVS assay, the wells were carefully emptied and washed three times with sterile distilled water to remove any unattached cells. The plates were then oven-dried at 45 °C for 15 min to fix the adherent cells. A 0.1% crystal violet solution (100 μL) was added to all wells, and the wells were incubated for 20 min at room temperature in the dark, with lids in place. After which, the plates were washed five times with running tap water to remove any excess or unabsorbed stain. Thereafter, the biofilm biomass was assessed semi-quantitatively by re-solubilizing the crystal violet stain bound to the adherent cells with 150 μL of 100% ethanol. The absorbance of the plates was read at 590 nm using a microplate reader (Epoch™ Microplate Spectrophotometer). The mean absorbance (OD_590_) of the sample was determined, and results were expressed as percentage inhibition using the equation below.

$$\%\;Inhibition=\frac{\left(OD\;negative\;control-OD\;media\;control)-(OD\;sample-OD\;sample\;control\right)}{\left(OD\;negative\;control-OD\;media\;control\right)}\times 100$$

##### Assessment of Biofilm Metabolic Activity/Viability of Biofilm Cells via INT Reduction Assay

The modified procedure described by Sandasi et al. (2010) and Klančnik et al. (2014) [62,63] was used to measure the inhibition of biofilm metabolic activity of the samples [30]. The [2-(4-iodophenyl)-3-(4-nitrophenyl)-5-phenyl-2*H*-tetrazolium] (INT) (Sigma, St. Louis, MO, USA) was dissolved in distilled water to a concentration of 1 mg/mL. After biofilm formation at different times (0, 24, and 48 h), as previously described in Section Anti-Biofilm Screening, samples and positive controls were added where necessary and incubated for 24 h at 37 °C. After incubation, the plates were washed three times, and 100 µL of INT was added to all wells. The plate was covered and incubated in the dark for 30 min. Afterwards, the absorbance was measured at 490 nm. The equation, as previously stated, was used to determine the percentage inhibition or reduction in biofilm metabolic activity by the samples.

### 3.4. Anti-Quorum-Sensing Assay

#### 3.4.1. Inhibition of Violacein Production

The anti-quorum-sensing activity of the extracts was evaluated in 48-well plates using the protocol of [30]. The bacterial suspension was prepared by inoculating a single colony of *Chromobacterium violaceum* from an agar plate into 10 mL LB broth and incubating for 24 h in an orbital shaker (140× *g*) at 30 °C before each experiment. To standardise the bacterial culture to obtain 1.5 × 10^6^ CFU/mL, corresponding to a McFarland No. 0.5 standard, a microplate reader was used at 590 nm. One mL of the overnight culture of *C. violaceum* was pipetted into a 200 mL sterile flask and diluted with 100 mL of LB broth. Then, 0.5 mL of fresh LB media was transferred into the wells, and 0.5 mg of extracts and positive control (gentamicin and ciprofloxacin) were added to their respective wells and serially diluted to obtain concentrations of 1.25 to 0.04 mg/mL and 0.25 to 0.008 mg/mL, respectively, except wells designated for blank (culture and media). Standard overnight culture (0.5 mL) was added to all wells. The plates were sealed adequately with parafilm and incubated in an orbital shaker (140× *g*) at 30 °C for 24 h. Following incubation, MICs were determined as the lowest concentration at which the samples inhibited growth and prevented purple pigmentation. The minimum quorum-sensing inhibitory concentration (MQSIC) was determined by the presence of growth (turbidity) with no visible purple pigmentation.

#### 3.4.2. Quantification of Violacein

The quorum quenching or quorum-sensing inhibition activity of the extracts was analysed using the procedure described by [30]. After quantifying violacein production, the plates were covered to prevent spilling, then centrifuged at 4000× *g* for 20 min to separate the bacterial pellet from the media. Afterwards, the supernatant (media) was removed, and the bacterial pellet was dissolved in 1 mL 100% dimethyl sulfoxide (DMSO) using an orbital shaker for 5 min. The mixture (200 mL) was transferred into the wells of a 96-well microplate, and the absorbance was measured at 595 nm to obtain the percentage of violacein inhibition calculated using the following formula.

[(ODcontrol − Odtest)/ODcontrol] × 100

### 3.5. Anti-Inflammatory Activities 0

#### 15-Lipoxygenase Inhibitory Assay

The anti-inflammatory activity was assessed by measuring the lipoxygenase inhibitory activity of the extracts, as described by Adebayo et al. (2015) [64]. The Tris-HCl buffer was prepared by dissolving 6.57 g of Tris powder in 800 mL of distilled water, and HCl was added gradually to the mixture to achieve a pH of 7.4. This buffer was used in the various reconstitutions of the extracts and the positive control. The substrate was prepared by mixing 43.2 µL of linoleic acid, 129.6 µL of 100% ethanol, 600 µL of Tween-80, and 200 mL of buffer together. One hundred µL of aliquoted 1 mg/mL 15-lipoxygenase (15-LOX) enzyme was added to 900 µL of buffer (stock). The ferrous oxidation-xylenol orange (FOX) reagents were prepared separately immediately before the experiment began. Xylenol orange (18.75 mg) was mixed with 90 mL of methanol and 10 mL of 300 mM sulphuric acid, while 3.79 mg of ferrous sulphate was mixed with 90 mL of methanol + 10 mL 300 mM sulphuric acid. A 200 unit/mL solution of 15-LOX was prepared and kept on ice while the assay was set up. Plant extracts were prepared (10 mg/mL) in DMSO and reconstituted to 2 mg/mL in the buffer. Quercetin (positive control) was prepared to 10 mg/mL in distilled water and reconstituted to 1 mg/mL in the buffer. The buffer (20 µL) was added to all wells. Afterwards, 20 µL of the various fractions was added only to the first well, then diluted in a serial manner. After this, 40 µL of the enzyme was added to all wells and incubated for 5 min at 37 °C. Then, 40 µL of the substrate was added to all wells except those designated as blanks, and the mixture was incubated for an additional 20 min. After 20 min of incubation, 100 µL of freshly prepared FOX mixture was added to the wells and incubated for an additional 30 min. Before reading the plates, 40 µL of the substrate was added to the blank wells. Using a microplate reader, absorbance was measured at 560 nm, and the results were calculated in percentage inhibition using the following formula.

$$\begin{array}{c} \%\;Enzyme\;activity=\frac{\left(Absorbance\;sample-Absorbance\;blank\right)}{\left(Absorbance\;negative\;control-Absorbance\;blank\right)}\times 100\\ \%\;Enzyme\;inhibition=100-\%\;enzyme\;activity \end{array}$$

### 3.6. Inhibition of Nitric Oxide (NO) Production in LPS-Induced RAW 264.7 Macrophages

The scavenging effect of the samples on LPS-induced cells was assessed as described by Yoon et al. (2009) [65] with modifications [66]. The ATCC RAW 264.7 macrophage cells were revived in Dulbecco’s Modified Eagle’s medium (DMEM) supplemented with 10% foetal bovine serum (Sigma, St. Louis, MO, USA) and 1% of penicillin (100 units/mL) and streptomycin (100 ug/mL) (Celtic Molecular Diagnostics, Cape Town, South Africa) at 37 °C in a 5% CO_2_ atmosphere (HERAcell 150, Thermo Fisher, Waltham, MA, USA). After that, the cells were seeded at a density of 4 × 10^4^ cells/well into each well of columns 2 to 11 of cell culture-treated, sterile 96-well plates (NEST, Whitehead Scientific, Cape Town, South Africa) and incubated for 24 h at 37 °C in 5% CO_2_. After 24 h of incubation, the media was carefully removed and replaced with fresh media. Lipopolysaccharide at 1 µg/mL was added to the wells, and 100 mL of the extracts at different concentrations (100–1.6 µg/mL) were used to treat the cells (except for the wells designated as blanks). The plates were incubated for an additional 24 h. After incubation, 100 μL from each incubated plate was transferred to a new plate, and 100 μL of Griess reagent (Sigma, St. Louis, MO, USA) was added. The plates were incubated for 15 min in the dark at room temperature (22 °C). The absorbance of the plates was measured at 540 nm using a microplate reader (Synergy HT, Biotek, Winooski, VT, USA). The sodium nitrite (NaNO_2_) standard curve was used to calculate the amount of nitrite in the media. The percentage of nitric oxide inhibition was calculated relative to the untreated LPS-induced cells.

#### Assessment of Cell Viability

The cell proliferation and cytotoxicity effects of the samples on RAW 264.7 cells were measured using the 3-(4,5-dimethylthiazolyl-2)-2,5-diphenyltetrazoliumbromide (MTT) colorimetric assay with slight modifications [67]. The breakdown of membrane-bound organelles by MTT to formazan was used to indicate the presence of viable cells. After incubating the plates for 24 h, the medium was aspirated from each well, and the plates were washed with 200 mL of pre-warmed phosphate-buffered saline (PBS). Afterwards, 100 mL of freshly prepared media was added to all wells, and 30 mL of MTT was also added to all the wells. The plates were then incubated in 5% CO_2_ at 37 °C for 4 h. After incubation, the medium was removed from all wells, while 50 mL of DMSO was added to dissolve the formazan salt precipitate. The absorbance was measured using a microplate reader at 570 nm. The percentage of viable cells in the area (wells) treated with the sample and the positive control was calculated relative to that of the untreated cells.

### 3.7. Cytotoxicity

The 3-(4,5-dimethylthiazolyl-2)-2.5-diphenyltetrazolium bromide (MTT) reduction assay was used to measure the viability of the cells after incubation with the test samples on bovine cells. The intensity of the colour (measured spectrophotometrically) of the MTT formazan yielded by metabolically active cells is proportional to the number of live cells present [67]. The cells of a sub-confluent culture were harvested and centrifuged at 200× *g* for 5 min and re-suspended in growth medium to 2.4 × 10^3^ cells/mL. The Minimal Essential Medium (MEM) supplemented with 0.1% gentamicin and 5% foetal calf serum was used as the growth medium. A total of 200 µL of the cell suspension was added to each well in columns 2–11 of a sterile 96-well microtitre plate. Exactly 200 µL of serum-free MEM was added to the wells of columns 1 and 12 to minimise the ‘edge effect’ and maintain humidity. Thereafter, plates were incubated for 24 h at 37 °C in a 5% CO_2_ incubator until the cells reached the exponential phase of growth. The MEM was aspirated from the cells, and 200 µL of test extracts at varying concentrations (prepared by serial dilution in growth medium) was added. The microtitre plates were incubated at 37 °C in a 5% CO_2_ incubator for 48 h with the test extracts and untreated cells, as well as a positive control (doxorubicin). After incubation, the MEM was removed, and the plates were washed with PBS. Fresh MEM (200 µL) was then added to each well. After this, 30 µL of MTT (from a stock solution of 5 mg/mL in PBS) was added to each well, and the plates were incubated for an additional 4 h at 37 °C. After 4 h incubation, the media was removed, and the cells on the plates were washed with PBS. The MTT formazan crystals were dissolved by adding 50 µL of DMSO to each well, and the plates were gently shaken until the MTT solution dissolved. Immediately thereafter, the amount of MTT reduction was measured by measuring absorbance in a microplate reader (at a wavelength of 570 nm). The absorbance of the wells in column 1, containing medium and MTT but no cells, was used to blank the plate reader. The LC_50_ values were calculated as the concentration of the test extract or positive control that resulted in a 50% reduction in absorbance compared to untreated cells (by linear regression).

The positive controls used for the different assays are used in veterinary medicine, which is the reason for their selection.

### 3.8. Statistical Analysis

The IC_50_ and LC_50_ values (concentration at which 50% inhibition occurred and 50% lethal concentration values, respectively) were determined using the linear and non-linear regression curves in Microsoft Excel. Each experiment was done in triplicate, and the values are presented as mean ± SEM. Percentages were also determined.

## 4. Conclusions

The novelty of the current study is that it provides the first comprehensive evidence that *Combretum elaeagnoides* leaf extracts, fractions, and the isolated flavonoid quercetin-3-*O*-rhamnoside possess multifunctional in vitro bioactivities against *Staphylococcus* species implicated in bovine mastitis. The methanol extract, particularly the ethyl acetate (EtOAc) and dichloromethane (DCM) fractions, demonstrated notable antibacterial activity against both clinical mastitis isolates and reference strains, with low MIC values and favourable selectivity indices, indicating selective toxicity toward bacterial cells rather than host cells.

Furthermore, significant inhibition of bacterial adhesion, biofilm formation, and biofilm metabolic activity was observed across different stages of biofilm development, highlighting their potential to disrupt established infections that are typically recalcitrant to conventional antibiotics. The strong anti-quorum-sensing activity, particularly of the CeEtOAc fraction, further underscores *C. elaeagnoides*’s capacity to attenuate bacterial communication and virulence without exerting strong bactericidal pressure, a strategy that may reduce the development of antimicrobial resistance.

Although the extracts exhibited pronounced anti-inflammatory activity by inhibiting nitric oxide production and 15-lipoxygenase activity, no significant differences were found between the samples. Importantly, the extracts and fractions were non-cytotoxic to bovine dermis cells, reinforcing their safety and potential suitability for veterinary applications. The authors recommended that further safety and pharmacokinetic experiments, as well as appropriate formulation and in vivo studies, be investigated.

## Figures and Tables

**Table 1:** Minimum Inhibitory Concentration (MIC) of *C. elaeagnoides* extracts in mg/mL.

	Organisms							
	SA1	SA2	SA3	SA4	SA5	SA6	SAA	SEA
MeOH	0.63 ± 0.00	0.52 ± 0.18	1.04 ± 0.36	0.62 ± 0.54	0.63 ± 0.00	0.63 ± 0.00	0.21 ± 0.09	0.83 ± 0.36
CeBuOH	0.42 ± 0.18	0.26 ± 0.09	0.34 ± 0.28	0.63 ± 0.00	0.26 ± 0.09	0.26 ± 0.09	0.23 ± 0.13	0.42 ± 0.18
CeDCM	0.11 ± 0.04	0.23 ± 0.13	0.42 ± 0.18	0.26 ± 0.09	0.26 ± 0.09	0.31 ± 0.00	0.11 ± 0.08	0.52 ± 0.18
CeH	0.21 ± 0.09	0.33 ± 0.30	0.52 ± 0.18	0.26 ± 0.09	0.31 ± 0.00	0.31 ± 0.00	0.16 ± 0.00	0.63 ± 0.00
CeAq	0.63 ± 0.00	0.62 ± 0.54	0.63 ± 0.00	0.63 ± 0.00	0.42 ± 0.18	0.94 ± 0.54	0.63 ± 0.00	0.73 ± 0.48
CeEtOAc	**0.16 ± 0.13**	**0.16 ± 0.13**	**0.21 ± 0.09**	**0.16 ± 0.00**	**0.12 ± 0.06**	**0.11 ± 0.05**	**0.07 ± 0.02**	**0.23 ± 0.13**
Q-3-*O*-rhamnoside	0.31 ± 0.00	0.31 ± 0.00	0.63 ± 0.00	1.25 ± 0.00	0.63 ± 0.00	0.63 ± 0.00	0.31 ± 0.00	0.63 ± 0.00
GENT	0.008	0.002	0.008	0.002	0.03	0.004	0.002	0.002

MeOH (methanol crude extract), CeBuOH (*Combretum elaeagnoides* butanol fraction), CeDCM (*Combretum elaeagnoides* dichloromethane fraction), CeH (*Combretum elaeagnoides* hexane fraction), (*Combretum elaeagnoides* water fraction), CeAq = *C. elaeagnoides* water fraction, CeEtOAc (*Combretum elaeagnoides* ethyl acetate fraction), GENT (Gentamicin) SA1–6 (clinical isolates of *S. aureus*), SAA and SEA (*S. aureus* ATCC 29213 [36], *S. epidermidis* ATCC 35984 [37]). Numbers in bold indicate promising activity.

**Table 2:** Anti-adhesion activity of *C. elaeagnoides* fractions and methanol extract at (T0) at 2 mg/mL.

Samples	Pathogens with Inhibition							
	SA1	SA2	SA3	SA4	SA5	SA6	SEA	SAA
MeOH	51.41 ± 0.11	29.66 ± 0.05	76.05 ± 0.02	38.19 ± 0.05	0.00	13.79 ± 0.02	**100.00**	62.71 ± 0.06
CeDCM	91.26 ± 0.03	61.06 ± 0.02	**100.00**	69.29 ± 0.02	42.86 ± 0.01	92.09 ± 0.02	**100.00**	92.32 ± 0.02
CeEtOAc	74.58 ± 0.14	78.20 ± 0.01	34.97 ± 0.01	76.59 ± 0.01	89.52 ± 0.01	73.84 ± 0.01	**100.00**	**100.00**
CeBuOH	72.38 ± 0.01	20.91 ± 0.08	0.00	3.64 ± 0.03	0.00	75.57 ± 0.01	**100.00**	79.56 ± 0.02
CeHx	87.78 ± 0.05	71.38 ± 0.04	87.63 ± 0.01	**100.00**	52.67 ± 0.03	87.74 ± 0.04	86.37 ± 0.04	**100.00**
CeAq	81.51 ± 0.01	46.96 ± 0.06	**100.00**	52.76 ± 0.05	0.00	80.98 ± 0.03	95.89 ± 0.01	98.16 ± 0.03
Ciprofloxacin	**100.00**	**100.00**	**100.00**	80.25 ± 0.01	91.68 ± 0.00	91.16 ± 0.00	95.27 ± 0.00	75.16 ± 0.01
Gentamicin	95.70 ± 0.00	82.79 ± 0.00	93.97 ± 0.01	76.40 ± 0.00	**100.00**	0.00	0.00	**100.00**

MeOH: methanol extract; CeDCM: *C. elaeagnoides* dichloromethane fraction; CeEtOAc: *C. elaeagnoides* ethyl acetate fraction; CeBuOH: *C. elaeagnoides* butanol fraction; CeHx: *C. elaeagnoides* hexane fraction; CeAq: *C. elaeagnoides* water fraction; T0: time zero; SA1–6 (clinical isolates of *S. aureus*); SAA and SEA (*S. aureus* ATCC 29213, *S. epidermidis* ATCC 35984). Numbers in bold indicate promising activity.

**Table 3:** Antibiofilm activity of *C. elaeagnoides* fractions and methanol extract at different times (T0, T24, T48) at 2 mg/mL.

Samples	Hour		% Inhibition						
		SA1	SA2	SA3	SA4	SA5	SA6	SEA	SAA
MeOH	T24	81.33 ± 0.14	35.09 ± 0.05	0.00	46.70 ± 0.15	7.28 ± 0.04	33.94 ± 0.50	46.76 ± 0.26	**100.00**
	T48	0.00	0.00	0.00	0.00	0.00	0.00	0.00	0.00
CeDCM	T24	91.07 ± 0.01	0.00	40.93 ± 0.01	64.67 ± 0.03	34.59 ± 0.06	68.81 ± 0.06	74.31 ± 0.17	38.78 ± 0.06
	T48	0.00	25.39 ± 0.08	36.71 ± 0.06	0.00	0.00	0.00	0.00	0.00
CeEtOAc	T24	82.69 ± 0.07	24.54 ± 0.02	0.00	81.45 ± 0.01	53.26 ± 0.06	43.56 ± 0.38	71.54 ± 0.23	56.84 ± 0.05
	T48	0.00	0.00	62.32 ± 0.14	0.00	0.00	0.00	0.00	0.00
CeBuOH	T24	67.84 ± 0.02	0.00	0.00	57.95 ± 0.03	0.00	75.64 ± 0.24	58.12 ± 0.12	13.11 ± 0.09
	T48	0.00	37.80 ± 0.20	42.64 ± 0.17	0.00	0.00	0.00	0.00	0.00
CeHx	T24	93.58 ± 0.02	1.53 ± 0.08	0.00	**100.00**	24.68 ± 0.02	44.07 ± 0.15	69.96 ± 0.45	36.92 ± 0.12
	T48	0.00	57.61 ± 0.16	68.78 ± 0.13	**100.00**	0.00	0.00	0.00	21.81 ± 0.03
CeAq	T24	0.00	0.00	0.00	0.00	0.00	0.00	75.88 ± 0.04	33.21 ± 0.07
	T48	0.00	0.00	0.00	0.00	0.00	0.00	0.00	0.00
Q-3-*O*-rhamnoside	T48	71.82 ± 0.13	0.00	75.97 ± 0.12	75.46 ± 0.08	0.00	0.00	**100.00**	76.24 ± 0.07
Ciprofloxacin	T24	97.35 ± 0.03	42.38 ± 0.02	95.92 ± 0.01	97.12 ± 0.01	41.55 ± 0.03	99.64 ± 0.01	0.00	88.73 ± 0.05
	T48	81.13 ± 0.31	8.52 ± 0.09	97.58 ± 0.01	0.00	**100.00**	76.83 ± 0.47	0.00	0.00
Gentamicin	T24	73.65 ± 0.01	36.03 ± 0.09	16.22 ± 0.11	74.10 ± 0.06	80.54 ± 0.05	96.68 ± 0.05	64.53 ± 0.10	86.50 ± 0.03
	T48	56.92 ± 0.49	54.03 ± 0.35	0.00	0.00	1.59 ± 0.38	55.70 ± 0.18	8.99 ± 0.16	44.93 ± 0.18

MeOH: methanol extract; CeDCM: *C. elaeagnoides* dichloromethane fraction; CeEtOAc: *C. elaeagnoides* ethyl acetate fraction; CeBuOH: *C. elaeagnoides* butanol fraction; CeHx: *C. elaeagnoides* hexane fraction; CeAq: *C. elaeagnoides* water fraction; T0: time zero; T24: 24 h; T48: 48 h; SA1–6 (clinical isolates of *S. aureus*); SAA and SEA (*S. aureus* ATCC 29213, *S. epidermidis* ATCC 35984). Numbers in bold indicate promising activity.

**Table 4:** Metabolic activity of the different fractions and the methanol extract of *C. elaeagnoides* (% inhibition).

Samples	Hour	% Inhibition							
		SA1	SA2	SA3	SA4	SA5	SA6	SAA	SEA
MeOH	T0	82.79 ± 0.05	**100.00**	99.80 ± 0.01	**100.00**	**100.00**	93.26 ± 0.01	**100.00**	34.95 ± 0.01
	T24	89.10 ± 0.00	76.69 ± 0.01	67.72 ± 0.19	**100.00**	85.07 ± 0.02	73.15 ± 0.03	72.81 ± 0.03	**100.00**
	T48	-	74.80 ± 0.01	38.63 ± 0.04	97.76 ± 0.08	90.55 ± 0.00	80.40 ± 0.22	**100.00**	**100.00**
CeDCM	T0	91.07 ± 0.01	**100.00**	68.43 ± 0.02	**100.00**	77.86 ± 0.00	90.40 ± 0.01	37.48 ± 0.03	**100.00**
	T24	50.00 ± 0.00	83.86 ± 0.03	77.82 ± 0.04	**100.00**	86.95 ± 0.00	60.47 ± 0.01	77.06 ± 0.00	51.25 ± 0.01
	T48	-	69.73 ± 0.01	76.04 ± 0.01	30.04 ± 0.02	82.62 ± 0.01	93.41 ± 0.03	86.34 ± 0.03	87.86 ± 0.03
CeEtOAc	T0	89.88 ± 0.02	**100.00**	**100.00**	**100.00**	78.64 ± 0.00	94.55 ± 0.00	**100.00**	**100.00**
	T24	60.53 ± 0.00	81.83 ± 0.01	78.84 ± 0.06	93.60 ± 0.00	**100.00**	68.29 ± 0.02	77.06 ± 0.01	90.66 ± 0.01
	T48	-	73.44 ± 0.00	72.90 ± 0.01	50.78 ± 0.01	69.18 ± 0.01	96.13 ± 0.01	86.34 ± 0.04	89.16 ± 0.01
CeBuOH	T0	88.48 ± 0.06	**100.00**	95.82 ± 0.03	**100.00**	99.03 ± 0.03	**100.00**	59.80 ± 0.06	31.32 ± 0.03
	T24	29.57 ± 0.00	73.03 ± 0.05	74.94 ± 0.05	81.63 ± 0.01	94.67 ± 0.01	73.15 ± 0.00	68.83 ± 0.02	86.99 ± 0.01
	T48	-	38.48 ± 0.00	60.71 ± 0.01	59.7 ± 0.04	61.93 ± 0.00	98.61 ± 0.01	47.17 ± 0.04	66.85 ± 0.01
CeHx	T0	60.27 ± 0.03	**100.00**	78.62 ± 0.02	57.5 ± 0.00	97.28 ± 0.00	96.56 ± 0.01	**100.00**	84.89 ± 0.00
	T24	60.53 ± 0.01	69.58 ± 0.06	58.68 ± 0.13	73.85 ± 0.01	69.85 ± 0.03	56.66 ± 0.02	89.72 ± 0.03	60.37 ± 0.01
	T48	-	3.78 ± 0.03	-	64.94 ± 0.03	40.52 ± 0.09	95.69 ± 0.01	-	61.53 ± 0.01
CeAq	T0	73.43 ± 0.02	**100.00**	89.71 ± 0.02	**100.00**	60.58 ± 0.01	96.27 ± 0.00	**100.00**	93.56 ± 0.00
	T24	29.70 ± 0.00	79.10 ± 0.01	74.20 ± 0.05	86.06 ± 0.00	81.97 ± 0.01	66.43 ± 0.01	65.14 ± 0.04	44.34 ± 0.01
	T48	**100.00**	32.42 ± 0.00	39.29 ± 0.02	84.01 ± 0.03	61.49 ± 0.01	96.85 ± 0.00	-	61.70 ± 0.01
Q-3-*O*-rhamnoside	T48	82.67 ± 0.00	74.08 ± 0.08	63.05 ± 0.08	98.56 ± 0.00	90.14 ± 0.00	81.69 ± 0.03	95.70 ± 0.03	86.81 ± 0.04
Gentamicin	T0	67.81 ± 0.00	61.02 ± 0.01	26.25 ± 0.10	100	38.99 ± 0.02	67.50 ± 0.08	81.77 ± 0.01	-
	T24	**100.00**	81.82 ± 0.00	47.94 ± 0.09	96.28 ± 0.01	**100.00**	**100.00**	3.66 ± 0.02	76.75 ± 0.03
	T48	85.77 ± 0.00	-	15.70 ± 0.10	80.83 ± 0.01	**100.00**	-	2.96 ± 0.04	11.55 ± 0.00
Ciprofloxacin	T0	**100.00**	-	15.70 ± 0.10	99.35 ± 0.05	98.48 ± 0.01	89.29 ± 0.02	13.86 ± 0.05	-
	T24	91.83 ± 0.00	87.56 ± 0.00	**100.00**	97.68 ± 0.00	71.62 ± 0.00	63.56 ± 0.01	66.57 ± 0.02	86.98 ± 0.03
	T48	-	32.61 ± 0.00	75.83 ± 0.01	52.79 ± 0.01	73.40 ± 0.01	68.41 ± 0.00	21.10 ± 0.01	-

CeEtOAc = *C. elaeagnoides* ethyl acetate fraction; CeBuOH = *C. elaeagnoides* butanol fraction; CeHx = *C. elaeagnoides* hexane fraction; CeAq = *C. elaeagnoides* water fraction; MeOH = methanol extract; CeDCM = *C. elaeagnoides* dichloromethane fraction; - = no inhibition; T0 = time zero; T24 = 24 h; T48 = 48 h; SA1–6 (clinical isolates of *S. aureus*); SAA and SEA (*S. aureus* ATCC 29213, *S. epidermidis* ATCC 35984). Numbers in bold indicate promising activity.

**Table 5:** Percentage of inhibition of violacein production in *C. violaceum* by *C. elaeagnoides* methanol extract and fractions.

	% Inhibition						MIC (mg/mL)	MQSIC (mg/mL)	MQSIC (Ic_50_ in mg/mL)
Conc. (mg/mL)	0.08	0.16	0.31	0.63	1.25	2.5			
MeOH	28.60	45.02	63.62	89.86	95.93	96.08	0.63	0.16	0.31
CeEtOAc	50.83	59.18	73.55	88.17	96.16	96.21	0.63	0.08	<0.08
CeBuOH	49.53	66.49	66.49	76.92	96.14	96.21	0.63	0.16	0.24
CeHx	40.72	40.72	53.11	77.57	87.27	96.08	-	0.31	0.31
CeAq	35.53	35.48	55.29	55.19	95.49	95.64	0.63	0.16	0.31
CeDCM	39.03	50.29	58.09	59.49	78.94	95.98	-	0.31	0.16
Ciprofloxacin	95.31	96.08	96.16	96.24	96.24	96.32	0.03	0.03	<0.03
Amphotericin B	94.99	96.06	96.16	96.24	96.29	96.29	0.03	0.03	<0.03
Gentamicin	75.03	90.82	94.27	95.18	96.29	96.27	0.25	0.03	<0.25

MQSIC = Minimum quorum-sensing inhibitory concentration; MIC = Minimum inhibitory concentration; CeEtOAc = *C. elaeagnoides* ethyl acetate fraction; CeBuOH = *C. elaeagnoides* butanol fraction; CeHx = *C. elaeagnoides* hexane fraction; CeAq = *C. elaeagnoides* water fraction; CeDCM = *C. elaeagnoides* dichloromethane fraction; - = no MIC; MeOH = methanol extract.

**Table 6:** Anti-inflammatory activity (% inhibition of NO) of the methanol extract and fractions of *Combretum elaeagnoides*.

Extracts	Nitric Oxide Inhibition (% NO inhibition) and % Viability of Macrophages (MTT Assay)								NO (IC_50_ µg/mL)	LOX (IC_50_ µg/mL)
	100 µg/mL		50 µg/mL		12.5 µg/mL		1.6 µg/mL			
	NO	MTT	NO	MTT	NO	MTT	NO	MTT		
MeOH	91.64 ± 5.17	100.80 ± 3.12	81.26 ± 18.33	97.41 ± 8.68	60.74 ± 46.32	96.30 ± 11.68	11.33 ± 19.67	95.00 ± 1.67	26.51	47.75
CeEtOAc	100.45 ± 6.48	100.40 ± 1.18	91.49 ± 11.31	100.91 ± 10.98	52.37 ± 16.89	100.56 ± 5.50	0.00	100.27 ± 3.58	8.19	17.57
CeBuOH	94.94 ± 11.74	98.02 ± 26.31	78.68 ± 32.45	92.44 ± 20.99	52.40 ± 58.16	97.89 ± 11.12	0.00	90.07 ± 20.90	7.57	22.04
CeHx	100.08 ± 3.61	94.09 ± 33.05	100.79 ± 2.95	98.43 ± 14.69	85.94 ± 3.95	98.65 ± 4.16	31.65 ± 5.41	93.05 ± 5.21	12.40	3.40
CeAq	99.69 ± 1.56	83.40 ± 5.69	92.36 ± 9.46	82.12 ± 8.32	77.32 ± 24.45	97.12 ± 0.55	27.40 ± 19.44	95.32 ± 5.15	23.59	4.94
CeDCM	100.80 ± 4.05	75.74 ± 19.16	91.66 ± 11.39	92.16 ± 8.84	62.36 ± 27.74	100.35 ± 0.43	0.00	100.44 ± 2.28	22.52	3.59
Quercetin	96.15 ± 0.8	31.52 ± 3.3	94.53 ± 4.9	54.99 ± 4.6	64.66 ± 5.6	81.1 ± 4.4	43.91 ± 3.5	99.00 ± 9.3	2.85	9.70
Doxo	-	31.52 ± 3.3	-	16.19 ± 7.1	-	63.52 ± 4.4	-	86.13 ± 5.6		

Doxo = Doxorubicin (conc µM/mL), - = Not applicable, CeEtOAc = *C. elaeagnoides* ethyl acetate fraction, CeBuOH = *C. elaeagnoides* butanol fraction, CeDCM = *C. elaeagnoides* dichloromethane fraction, CeHx = 0 *C. elaeagnoides* hexane fraction, CeAq = *C. elaeagnoides* water fraction, MeOH = methanol extract.

**Table 7:** LC_50_ values against bovine dermis cells and selectivity index (LC_50_/MIC) of the methanol extract and fractions of *Combretum elaeagnoides* in mg/mL.

Extracts	LC_50_ (mg/mL)	Selectivity Index							
		SA1	SA2	SA3	SA4	SA5	SA6	SAA	SEA
MeOH	1	1.59	1.92	0.96	1.61	1.59	1.59	4.76	1.20
CeEtOAc	0.88	5.5	5.5	3.83	3.83	3.83	3.83	3.83	3.83
CeBuOH	1	2.38	3.85	2.94	1.59	3.85	3.85	4.35	2.38
CeHx	1	4.76	3.03	1.92	3.85	3.23	3.23	6.25	1.59
CeAq	1	1.59	1.61	1.59	1.59	2.38	1.06	1.59	1.37
CeDCM	1	9.09	4.35	2.38	3.85	3.85	3.23	9.09	1.92
Doxorubicin	0.008 ± 0.00	-	-	-	-	-	-	-	-

MeOH = methanol extract, - = Not applicable, CeEtOAc = *C. elaeagnoides* ethyl acetate fraction, CeBuOH = *C. elaeagnoides* butanol fraction, CeDCM = *C. elaeagnoides* dichloromethane fraction, CeHx = 0 *C. elaeagnoides* hexane fraction, CeAq = *C. elaeagnoides* water fraction. SA1–6 (clinical isolates of *S. aureus*); SAA and SEA (*S. aureus* ATCC 29213, *S. epidermidis* ATCC 35984).

## Data Availability

The original contributions presented in this study are included in the article. Further inquiries can be directed to the corresponding author.

## References

[1] Karzis J., Petzer I.-M., Naidoo V., Donkin E.F. (2021). The spread and antimicrobial resistance of *Staphylococcus aureus* in South African dairy herds—A review. Onderstepoort J. Vet. Res..

[2] Gomes F., Henriques M. (2016). Control of bovine mastitis: Old and recent therapeutic approaches. Curr. Microbiol..

[3] Balemi A., Gumi B., Amenu K., Girma S., Gebru M., Tekle M., Ríus A.A., D’Souza D.H., Agga G.E., Kerro Dego O. (2021). Prevalence of mastitis and antibiotic resistance of bacterial isolates from cmt positive milk samples obtained from dairy cows, camels, and goats in two pastoral districts in southern Ethiopia. Animals.

[4] Cheng W.N., Han S.G. (2020). Bovine mastitis: Risk factors, therapeutic strategies, and alternative treatments—A review. Asian-Australas. J. Anim. Sci..

[5] Macías Alonso M., López Salazar J.C., Osegueda Robles S., Córdova Guerrero I., Ledezma García F., Marrero J.G. (2020). In vitro antimicrobial activity of Mexican plants on bovine mastitis bacteria: Preliminary studies. Biosci. J..

[6] Harvard Health (2020). Understanding Acute and Chronic Inflammation.

[7] Hansen P.J., Soto P., Natzke R.P. (2004). Mastitis and fertility in cattle—Possible involvement of inflammation or immune activation in embryonic mortality. Am. J. Reprod. Immunol..

[8] Cobirka M., Tancin V., Slama P. (2020). Epidemiology and Classification of Mastitis. Animals.

[9] Pascu C., Herman V., Iancu I., Costinar L. (2022). Etiology of mastitis and antimicrobial resistance in dairy cattle farms in the western part of Romania. Antibiotics.

[10] Dadgostar P. (2019). Antimicrobial resistance: Implications and costs. Infect Drug Res..

[11] Erhabor R.C., Erhabor J.O., Nkadimeng S.M., McGaw L.J. (2022). In vitro antimicrobial, antibiofilm and antioxidant activities of six South African plants with efficacy against selected foodborne pathogens. S. Afr. J. Bot..

[12] Abebe G.M. (2020). The role of bacterial biofilm in antibiotic resistance and food contamination. Int. J. Microbiol..

[13] Foster T.J. (2017). Antibiotic resistance in *Staphylococcus aureus*. Curr. Opin. Microbiol..

[14] Chambers H.F., DeLeo F.R. (2009). Waves of resistance in *Staphylococcus aureus*. Nat. Rev. Microbiol..

[15] Davies J., Davies D. (2010). Origins and evolution of antibiotic resistance. Microbiol. Mol. Biol. Rev..

[16] Costa S.S., Viveiros M., Amaral L., Couto I. (2013). Multidrug efflux pumps in *Staphylococcus aureus*: An update. Open Microbiol. J..

[17] Munita J.M., Arias C.A. (2016). Mechanisms of antibiotic resistance. Microbiol. Spectr..

[18] Reygaert W.C. (2018). An overview of the antimicrobial resistance mechanisms of bacteria. AIMS Microbiol..

[19] Martini N.D., Eloff J.N. (1998). The preliminary isolation and of several antibacterial compounds from *Combretum erythrophyllum* (Combretaceae). J. Ethnopharmacol..

[20] Aderogba M.A., Kgatle D.T., McGaw L.J., Eloff J.N. (2012). Isolation of antioxidant constituents from *Combretum apiculatum* subsp. *apiculatum*. S. Afr. J. Bot..

[21] Erhabor R.C., Erhabor J.O., McGaw L.J. (2019). The potential of South African medicinal plants against microbial biofilm and quorum sensing of foodborne pathogens: A review. S. Afr. J. Bot..

[22] Erhabor R.C., Aderogba M.A., Erhabor J.O., Nkadimeng S.M., McGaw L.J. (2021). In vitro bioactivity of the fractions and isolated compound from *Combretum elaeagnoides* leaf extract against selected foodborne pathogens. J. Ethnopharmacol..

[23] Khunoana E.T., Eloff J.N., Ramadwa T.E., Nkadimeng S.M., Selepe M.A., McGaw L.J. (2022). In vitro antiproliferative activity of *Ptaeroxylon obliquum* leaf extracts, fractions and isolated compounds on several cancer cell lines. Appl. Sci..

[24] Akinboye A.O., Famuyide I.M., Petzer I.-M., McGaw L.J. (2023). In vitro antibacterial activity of selected South African plants against drug-resistant staphylococci isolated from clinical cases of bovine mastitis. Appl. Sci..

[25] Jambwa P., Makhubu F.N., Matope G., Fouche G., McGaw L.J. (2022). Bioassay guided fractionation of *Senna singueana* and its potential for development of poultry phytogenic feed additives. Front. Vet. Sci..

[26] McGaw L.J., Rabe T., Sparg S.G., Jager A.K., Eloff J.N., van Staden J. (2001). An investigation on the biological activity of *Combretum* species. J. Ethnopharmacol..

[27] Eloff J.N., Katerere D.R., McGaw L.J. (2008). The biological activity and chemistry of the southern African Combretaceae. J. Ethnopharmacol..

[28] Jordaan M., Van Wyk A.-E., Maurin O. (2011). A conspectus of *Combretum* (Combretaceae) in southern Africa, with taxonomic and nomenclatural notes on species and sections. Bothalia.

[29] Masoko P., Picard J., Eloff J.N. (2007). The antifungal activity of twenty-four southern African *Combretum* species (Combretaceae). S. Afr. J. Bot..

[30] Erhabor R.C., Erhabor J.O., Nkadimeng S.M., Petzer I.-M., Dzoyem J.-P., McGaw L.J. (2024). In vitro biological activities of *Combretum molle* R.Br.ex G.Don (Combretaceae) against mastitis-causing organisms. S. Afr. J. Bot..

[31] Hyde M.A., Wursten B.T., Ballings P., Coates Palgrave M. (2021). Flora of Zimbabwe: Information About the Species Data Pages. https://www.zimbabweflora.co.zw/speciesdata/about.php.

[32] Chinsembu K.C. (2016). Ethnobotanical study of plants used in the management of HIV/AIDS-related diseases in Livingstone, Southern Province, Zambia. Evid. Based Complement. Altern. Med..

[33] Chinsembu K.C., Syakalima M., Semenya S.S. (2019). Ethnomedicinal plants used by traditional healers in the management of HIV/AIDS opportunistic diseases in Lusaka, Zambia. S. Afr. J. Bot..

[34] Tolosa T., Wagaye H., Regassa F. (2009). A Study on in vitro antimicrobial effects of some selected plants on *Staphylococcus aureus* isolated from bovine clinical mastitis. Internet J. Vet. Med..

[35] Regassa F., Araya M. (2012). In vitro antimicrobial activity of *Combretum molle* (Combretaceae) against *Staphylococcus aureus* and *Streptococcus agalactiae* isolated from crossbred dairy cows with clinical mastitis. Trop. Anim. Health Prod..

[36] ATCC 29213. https://www.atcc.org/products/29213.

[37] ATCC 35984. https://www.atcc.org/products/35984.

[38] ATCC 39183. https://www.atcc.org/products/39183.

[39] ATCC 13076. https://www.atcc.org/products/13076.

[40] ATCC 25922. https://www.atcc.org/products/25922.

[41] ATCC 33560. https://www.atcc.org/products/33560.

[42] Orlandi V.T., Bolognese F., Chiodaroli L., Armenia I., Caruso E., Malacarne M.C. (2024). Antibiofilm activity of *Combretum micranthum* G. Don Catechin-Sugar Phytocomplex on *Pseudomonas aeruginosa*. Molecules.

[43] Relucenti M., Familiari G., Donfrancesco O., Taurino M., Li X., Chen R., Artini M., Papa R., Selan L. (2021). Microscopy methods for biofilm imaging: Focus on SEM and VP-SEM Pros and Cons. Biology.

[44] Adeyemo R.O., Famuyide I.M., Dzoyem J.-P., McGaw L.J. (2022). Anti-biofilm, antibac-terial, and anti-quorum sensing activities of selected South African plants traditionally used to treat diarrhoea. Evid. Based Complement. Altern. Med..

[45] Bowden G.H., Li Y.H. (1997). Nutritional influences on biofilm development. Adv. Dent. Res..

[46] Salgar-Chaparro S.J., Lepkova K., Pojtanabuntoeng T., Darwin A., Machuca L.L. (2020). Nutrient level determines biofilm characteristics and the subsequent impact on microbial corrosion and biocide effectiveness. Appl. Environ. Microbiol..

[47] Rutherford S.T., Bassler B.L. (2012). Bacterial quorum sensing: Its role in virulence and possibilities for its control. Cold Spring Harb. Perspect. Med..

[48] Vattem D.A., Mihalik K., Crixell S.H., McLean R.J. (2007). Dietary phytochemicals as quorum sensing inhibitors. Fitoterapia.

[49] Ogbuadike E.C., Nkadimeng S.M., Igwe C.C., Dzoyem J.-P., Qekwana D.N., Petzer I.-M., McGaw L.J. (2023). An invitro study on the potential of selected South African plant extracts to prevent and treat bovine mastitis. S. Afr. J. Bot..

[50] Singh N.K., Rao G.N. (2019). Emerging role of 12/15-Lipoxygenase (ALOX15) in human pathologies. Prog. Lipid Res..

[51] Mohamed R., Sullivan J.C. (2023). Sustained activation of 12/15 lipoxygenase (12/15 LOX) contributes to impaired renal recovery post ischemic injury in male SHR compared to females. Mol. Med..

[52] Adebayo S.A., Ondua M., Shai L.J., Lebelo S.L. (2019). Inhibition of nitric oxide production and free radical scavenging activities of four South African medicinal plants. J. Inflamm. Res..

[53] Angeh J.E., Huang X., Sattler I., Swan G.E., Dahse H., Härt A., Eloff J.N. (2007). Antimicrobial and anti-inflammatory activity of four known and one new triterpenoid from *Combretum imberbe* (Combretaceae). J. Ethnopharmacol..

[54] Mbiantcha M., Almas J., Dawe A., Faheem A., Sidra Z. (2018). Analgesic, anti-inflammatory and anticancer activities of Combretin A and Combretin B isolated from *Combretum fragrans* F. HOFFM (Combretaceae) leaves. Inflammopharmacology.

[55] Selogatwe K.M., Asong J.A., Struwig M., Ndou R.V., Aremu A.O. (2021). A review of ethnoveterinary knowledge, biological activities and secondary metabolites of medicinal woody plants used for managing animal health in South Africa. Vet. Sci..

[56] Masuku N.P., Erhabor R.C., Mokoka T.A., Ramagoma R.B., Maqolo K., McGaw L.J. (2025). South African medicinal plants as natural defences against bovine mastitis: A review. S. Afr. J. Bot..

[57] Zirihi G.N., Mambu L., Guede-Guina F., Bodo B., Grellier P. (2005). In vitro antiplasmodial activity and cytotoxicity of 33 West African plants used for treatment of malaria. J. Ethnopharmacol..

[58] Shai L., McGaw L.J., Masoko S., Eloff J.N. (2008). Antifungal and antibacterial activity of seven traditionally used South African plant species active against *Candida albicans*. S. Afr. J. Bot..

[59] Makhafola T.J., Eloff J.N. (2012). Five Ochna species have high antibacterial activity and more than 10 antibacterial compounds. S. Afr. J. Sci..

[60] Mphahlele M.P., Oguttu J.W., Petzer I.-M., Qekwana D.N. (2020). Prevalence and antimicrobial drug resistance of *Staphylococcus aureus* isolated from cow milk samples. Vet. World.

[61] Eloff J.N. (1998). A sensitive and quick method to determine the minimum inhibitory concentration of plant extracts for bacteria. Planta Medica.

[62] Sandasi M., Leonard C.M., Viljoen A.M. (2010). The in vitro anti-biofilm activity of selected culinary herbs and medicinal plants against *Listeria monocytogenes*. Lett. Appl. Microbiol..

[63] Klančnik A., Podobnik P., Možina S.S., Raspor P., Bohinc K., Jeršek B. Determination of viable biofilm cells in microtiter plates. Proceedings of the II International Congress, Food Technology, Quality and Safety.

[64] Adebayo S.A., Dzoyem J.P., Shai L.J., Eloff J.N. (2015). The anti-inflammatory and antioxidant activity of 25 plant species used traditionally to treat pain in southern Africa. BMC Complement. Altern. Med..

[65] Yoon W.-J., Kim S.-S., OH T.-H., Lee N.H. (2009). *Cryptomeria japonica* essential oil inhibits the growth of drug-resistant skin pathogens and LPS-Induced nitric oxide and Pro-Inflammatory cytokine production. Pol. J. Microbiol..

[66] Lawal F., Bapela M.J., Adebayo S.A., Nkadimeng S.M., Yusuf A.A., Malterud K.E., McGaw L.J., Tshikalange T.E. (2019). Anti-inflammatory potential of South African medicinal plants used for the treatment of sexually transmitted infections. S. Afr. J. Bot..

[67] McGaw L.J., Steenkamp V., Eloff J.N. (2007). Evaluation of *Athrixia* bush tea for cytotoxicity, antioxidant activity, caffeine content and presence of pyrrolizidine alkaloids. J. Ethnopharmacol..

